# Long-term evaluation of the rise in legal age-of-sale of cigarettes from 16 to 18 in England: a trend analysis

**DOI:** 10.1186/s12916-020-01541-w

**Published:** 2020-04-08

**Authors:** Emma Beard, Jamie Brown, Sarah Jackson, Robert West, Will Anderson, Deborah Arnott, Lion Shahab

**Affiliations:** 1 Department of Behavioural Science and Health, University College London, 1-19 Torrington Place, London, UK; 2 Action on Smoking and Health (ASH), London, UK

**Keywords:** Smoking, Youth, Age-of-sale, Tobacco

## Abstract

**Objective:**

To assess the long-term impact of the increase in age-of-sale of cigarettes from 16 to 18 in England in October 2007.

**Methods:**

Data were collected between November 2006 and September 2018 on 252,601 participants taking part in a nationally representative survey of adults aged 16+ in England, the Smoking Toolkit Study (STS). We assessed the impact of the introduction of the increase in age-of-sale on prevalence of ever smoking, current smoking, and quit attempts, among 16–17 year olds compared with 18–24 year olds.

**Results:**

Following the increase in age-of-sale, there was a declining trend in ever smoking that was greater among 16–17 year olds than 18–24 year olds (OR 0.990 versus OR 0.993; *p* = 0.019). Data on current smoking were insensitive to detect a difference between the age groups in a step-level change or change in trend following the increase in age-of-sale (Bayes factors (BFs) 0.75 and 2.10). Data on quit attempts were also insensitive to detect a change in trend (BF 0.71), and despite a greater step-level decline among those aged 16–17 (OR 0.311 versus OR 0.547, *p* = 0.025), quit attempts remained higher among those aged 16–17. Secondary analysis indicated that post-policy change, trends in current and ever smoking were linear for 16–17 year olds but quadratic for 18–24 year olds (slowing decline).

**Conclusion:**

There is some evidence from an assessment of long-term trends in the Smoking Toolkit Study that the increase in legal age-of-sale of cigarettes in England was associated with a greater long-term decline in ever smoking among those aged 16–17 compared with those aged 18–24.

## Introduction

A majority of smokers in the UK report that they began smoking before the age of 18, with the mean age of onset of regular smoking being 16 years [1]. In England, the legal age-of-sale was increased from 16 to 18 years on 1 October 2007 [2]. This does not apply to consumption, with tobacco products only confiscated by the police for those under 16 years of age. There have been calls in the UK to raise the legal age-of-sale further to 21 [3], the hope being that this will not only prevent uptake but reduce teenagers’ access to tobacco products purchased by their peers. Other jurisdictions have already increased the legal age-of-sale to 21, and there is evidence for its effectiveness [4, 5]. This paper considers the impact of the increase in age-of-sale restrictions in England from 16 to 18 on long-term trends in current smoking, ever smoking (as a measure of uptake), and attempts to quit among young people. Such findings will help to inform policymakers as to the possible effectiveness of increasing the legal age-of-sale further [3].

Two studies have formally assessed the short-term effects of the age-of-sale increase in England, showing that there was an immediate fall in youth smoking rates [2, 6]. However, these evaluations had several limitations. In the study by Fidler and West [2], a simple pre-post comparison was made, with an assessment of moderation effects by age. Thus, it was not possible to account for underlying secular trends, seasonal effects, or the introduction of other population-level tobacco control polices. This sort of analysis also precludes the ability to assess non-linear trends triggered by the introduction of the increase in age-of-sale. Although Millett et al. [6] looked at changes over time and accounted for secular trends, their analysis focused solely on 11 to 15 year olds meaning it was not possible to test for differences between age groups for whom buying cigarettes did or did not remain legal. They also did not adjust for population-level policies or seasonality, and the annual nature of data collection meant it was not possible to do a more fine grained analysis in the months immediately after the rise in age-of-sale.

Descriptive and quasi-experimental evidence from outside of the UK also suggests that short- to long-term effects are likely to arise [7]. In Sweden, compliance, assessed through test purchasing, increased over an 8-year period following the introduction of the minimum age of purchase [8]. These findings were supported in a survey of 19 European Union countries, which showed that laws prohibiting the sales of tobacco to minors were associated with lower perceived obtainability of cigarettes [9]. In a state-level study in the USA, cigarette control laws were shown to be effective in reducing the rate of cigarettes sold by merchants to minors [10], while after the tobacco sales ban to minors in 1995 in Finland, there was a permanent decline in purchases from commercial sources among those aged 14–16 [11]. A study in Canada assessed the impact of ‘hypothetical’ changes in the legal age-of-sale on smoking prevalence, finding that both daily and non-daily smoking was much higher in those older than the minimum age-of-sale [12].

This study aimed to conduct the first formal analysis of the long-term impact on youth smoking of the increase in age-of-sale of cigarettes from 16 to 18 years in England in October 2007. Monthly data were used from the Smoking Toolkit Study (STS) (www.smokinginengland.info), a population-level survey of adults age 16+ in England, between November 2006 and September 2018. We assessed the differential impact of the introduction of the increase in age-of-sale on prevalence of current smoking, ever smoking, and quit attempts among 16–17 year olds and 18–24 year olds while adjusting for underlying trends, seasonality, and population-level policies introduced during that period. Both linear and non-linear trends were modelled. Previous studies have generally focused on uptake of smoking or smoking prevalence. However, population level time series analyses provide preliminary evidence that the rise in age-of-sale may also have had an impact on quitting activity, at least in the short term [13, 14].

## Methods

### Design

Data were used from the STS between November 2006 and September 2018. The STS is a monthly survey of a representative sample of the population in England aged 16+, which collects data on smoking patterns among smokers and recent ex-smokers [15]. The STS uses a random location sampling design, with initial random selection of grouped output areas (containing 300 households), stratified by ACORN (socio-demographic) characteristics (http://www.caci.co.uk/acron/acornmap.asp) and region. Interviewers then choose which houses within these areas are most likely to fulfil their quotas and conduct face-to-face computer-assisted interviews with one member per household. The STS sample appears to be representative of the population in England, having a similar socio-demographic composition to other large national surveys, such as the Health Survey for England and retail sales data [15, 16].

### Measures

#### Outcomes

Participants were asked: ‘Which of the following best applies to you? a) I smoke cigarettes (including hand-rolled) every day; b) I smoke cigarettes (including hand-rolled), but not every day; c) I do not smoke cigarettes at all, but I do smoke tobacco of some kind (e.g. pipe or cigar); d) I have stopped smoking completely in the last year; e) I stopped smoking completely more than a year ago; f) I have never been a smoker (i.e. smoked for a year or more)’. Current smokers were defined as those who reported a, b, or c and ever smokers as those who reported a, b, c, d, or e.

Past-year smokers (a, b, c, or d) were also asked: ‘How many serious attempts to stop smoking have you made in the last 12 months? By serious attempt I mean you decided that you would try to make sure you never smoked again. Please include any attempt that you are currently making and please include any successful attempt made within the last year’. Past-year quit attempts were coded 1 for participants who reported having made at least 1 serious quit attempt and 0 for those who did not report any serious quit attempts in the last 12 months.

#### Seasonality, trends, and intervention effects

We included two time variables to account for underlying secular trends (coded 1…*n*, where *n* is the total number of waves) and seasonality (with months coded 0 through 11). An additional variable reflected the pre- and post-implementation periods in order to identify step-level changes in prevalence (coded 0 before the increase in age-of-sale and 1 after). Finally, we included a variable to reflect the change in trend following the increase in age-of-sale (coded 0 before the intervention and 1…*m* after the intervention, where *m* is the number of waves after the increase in age-of-sale).

#### Covariates

Participants were asked for other socio-demographic information (including age, gender, and social grade). Social grade was measured using the British National Readership Survey (NRS) Social-Grade Classification Tool, which on the basis of the occupation of the chief income earner in the household categorises people as ABC1 (this included managerial, professional, and intermediate occupations) versus C2DE (this includes small employers, and own-account workers, lower supervisory and technical occupations, and semi-routine and routine occupations, never workers, and long-term unemployed) [17].

Monthly tobacco control mass media expenditure (in million £) was obtained from Public Health England. The following tobacco control policies were also adjusted for using a temporary pulse effect, whereby a dummy variable was coded 0 before and after the policy was introduced and 1 during the month the policy was introduced: (1) the introduction of a ban in enclosed public spaces in July 2007 [18], (2) pictorial health warnings on product packaging introduced in October 2008 [19], (3) licencing of NRT for harm reduction in December 2009 [20], (4) point-of-sale ban introduced in England in April 2012 [21], (5) the move in commissioning of stop smoking services to local authorities in April 2013 [22], and (6) the publication of NICE guidance on harm reduction in June 2013 [23]. Sensitivity analyses were also run which modelled the two policies occurring closest in time to the increase in age-of-sale, the introduction of a ban in enclosed public spaces and the pictorial health warnings on product packaging, as long-term step-level changes.

Finally, sensitivity analyses adjusted for self-reported average weekly spend on tobacco. To assess expenditure on smoking, current smokers were asked: ‘On average about how much per week do you think you spend on cigarettes or tobacco?’ and to report the number of cigarettes they smoke per day. Smokers’ average cost of smoking (in £/week) was derived from the following liberal assumptions for upper estimates of plausible levels of consumption and expenditure per week: (1) smokers smoke a maximum of 560 cigarettes per week, (2) spending does not exceed £280 per week, and (3) single cigarettes cost between £0.05 and £1 [24].

These covariates were chosen a priori and included in the pre-registered analysis plan. Decisions were based on previous research showing their association with smoking uptake and quitting activity. For example, the introduction of a ban in enclosed public spaces in July 2007 was found to be associated with a temporary increase in the percentage of smokers attempting to stop [18], while pictorial health warnings on product packaging introduced in October 2008 have been found to promote smoking cessation and may reduce uptake [19, 25]. An evaluation of the partial (i.e. supermarket) tobacco point-of-sale display ban introduced in England in April 2012 found evidence for a decline in smoking prevalence [21]. Licencing of nicotine replacement therapy for use in harm reduction, and higher expenditure on tobacco control mass media have been found to be associated with the success of quit attempts [26]. Time series analyses have also shown that cuts on tobacco mass media expenditure have been associated with a reduction in use of smoking cessation support [27, 28]. Smoking prevalence is highest among those from disadvantaged backgrounds, and these smokers appear less likely to succeed in qutting than those from more advantaged groups [29, 30]. There are also gender differences in smoking, with men tending to use tobacco products at higher rates than women [31]. Finally, tax increases over the study period have resulted in the rise in cost of smoking [32], and although smokers often adjust their behaviour to account for these, tax increases have been shown to be a cost-effective intervention for reducing smoking prevalence by promoting quitting and reducing uptake [33, 34].

### Analysis

Questions on the cost of cigarettes were only collected between October 2007 and June 2009 and then from August 2010 onwards. Missing values for these periods were imputed using Kalman Smoothing for univariate time series data [35].

Data were analysed in R studio. The analysis plan was pre-registered on the Open Science Framework (https://osf.io/3wgmn/). First, unweighted and weighted socio-demographic and smoking descriptive statistics were calculated. The STS uses rim (marginal) weighting to match the sample to the population in England on the dimensions of age, social grade, region, housing tenure (bought on a mortgage, owned outright, rented from local authority, and rented from private landlord), ethnicity, and working status (working or not working) within sex.

#### Primary analysis—difference-in-differences

Generalised Additive Models (GAMs) were used for the difference-in-differences (DID) segmented analysis at the individual non-aggregated level. These allow the fitting of seasonal smoothing terms and thus seasonality to be taken into account. For the primary analysis, we compared 16–17 year olds with 18–24 year olds. The unadjusted DID model was specified as:

$$ {y}_t={\beta}_0+{\beta}_1\mathrm{age}+{\beta}_2\mathrm{trend}+{\beta}_3{\mathrm{level}}_t+{\beta}_4\mathrm{slope}+{\beta}_5{\mathrm{level}}_t\times \mathrm{age}+{\beta}_6\mathrm{slope}\times age+{e}_t $$

*y*_*t*_ reflects the dependent variable at time *t* (i.e. prevalence), *β* reflects the various coefficients, and *e*_*t*_ is the error at time *t*. Level distinguishes the periods pre- and post-implementation of the increase in age-of-sale, coded 0 before the increase and 1 after. Trend accounts for secular trends with a variable coded 1…*n*, where *n* is the number of months in the study period. Slope reflects the slope after the introduction of the increase in age-of-sale coded 0 before the introduction and 1…*m* after, where *m* is the number of months in the study period after the increase in age-of-sale. Additional models were run adjusting for gender, social grade (ABC1 versus C2DE), tobacco control mass media spend, and population-level policies occurring during the study period. For the primary analysis, a linear underlying and post-implementation trend was assumed.

#### Secondary analyses—trend analysis

Secondary analyses then assessed whether polynomial regression with terms up to an order of 3 (i.e. quadratic trend and cubic trend model) for the post-implementation trend provided a better fit for 16–17 and 18–24 year olds. This was then repeated with segmented regression models. These allow relationships that are linear but segmented, namely represented by at least two lines connected at ‘breakpoints’. Breakpoints were determined using an iterative procedure, whereby models with different positions of maximally one breakpoint were compared using the Akaike information criterion (AIC). The best fitting model was chosen as the model with lowest AIC. Generally, the simplest model is chosen where the difference in AIC (delta) is < 2.

Bayes factors (BFs) were derived for the primary non-significant findings using an online calculator to disentangle whether there was evidence for the null hypothesis of no effect (BF < 1/3), the alternative hypothesis (BF > 3) or whether data were insensitive (e.g. large standard error or lack of power) [36]. A half-normal distribution was assumed with an effect size for a step-level change of OR = 1.36 and for the change in slope of OR = 1.001. These were derived from a study assessing the short-term effects of increase of age-of-sale from 16 to 18 [2].

STrengthening the Reporting of Observational studies in Epidemiology (STROBE) guidelines were followed throughout [37].

#### Sensitivity analysis

Sensitivity analyses repeated the primary analysis with the older age group split into 18–21 year olds and 22–24 year olds. This is important given the argument that the increase in age-of-sale should be increased further to 21 [3].

#### Amendments to the analysis plan

An amendment was made to the analysis plan in May 2019. As the underlying trend did not differ significantly as a function of age group, an interaction term between the underlying trend and age was not included in the final model to preserve power. The underlying trend before the increase in age-of-sale was thus assumed to be the same among 16–17 year olds and 18–24 year olds. The fitted trend was found to fit the pre-implementation data well for both age groups.

An additional amendment was made in June 2019 to the analysis plan following calculation of Bayes factors which suggested that the data were insensitive to detect a difference among 16–17 year olds and 18–24 year olds in a step-level change and change in trend. An additional age group (25+ years) was added to the primary and secondary analyses. Although this increased the power to detect a difference in step-level change and change in trend, the pre-implementation modelled trend for 16–17 year olds was dominated by the 25+ age group and thus proved a poor fit despite no evidence for a significant difference in the pre-intervention slope across the three groups for either smoking status, ever smoking status, or attempts to quit smoking (*p* = 0.660 to 0.875). Thus, the decision was made to include the results from this comparison only as supplementary material. Additional file 1: Tables S1-S5 give the results for those aged 25+ relative to 16–17 year olds and mirror Tables 1, 2, 3, 4, and 5 for 18–24 year olds relative to 16–17 year olds.

**Table 1:** Descriptive statistics of the sample overall and as a function of the two age groups of interest (16–17 year olds and 18–24 year olds)

	Overall (*n* = 252,601) Unweighted, % (*n*) Weighted, % (*n*)	16–17 year olds (*n* = 5190) Unweighted, % (*n*) Weighted, % (*n*)	18–24 year olds (*n* = 30,681) Unweighted, % (*n*) Weighted, % (*n*)
Gender			
Female	51.64 (130,434)	42.91(2227)^a^	47.35(14,526)^b^
	51.20 (129,327)	44.96 (2599)	49.21 (15,839)
Other	48.36 (122,167)	57.09(2962)	52.65(16,155)
	48.80 (123,274)	55.04 (3182)	50.79 (16,349)
Social grade			
ABC1	60.72 (178,191)	64.89 (3369)^a^	67.06(20,576)^b^
	76.57 (193,419)	71.06 (4108)	71.19 (22,916)
C2DE	29.46 (74,410)	35.09 (1821)	32.94(10,105)
	23.43 (59,183)	28.94 (16773)	28.81 (9272)
Current smoker	21.33 (53,831)	15.59(809)^a^	28.73(8808)^b^
	20.58 (51,957)	15.54 (898)	27.91 (8975)
Ever smoker	37.66 (95,060)	17.81(924)^a^	34.07(10,443)^b^
	36.68 (92,577)	17.87 (1033)	33.30 (10,711)
Quit attempt^¥^	35.87 (20,238)	44.38(379)^a^	39.91(3694)^b^
	36.15 (19,877)	43.94 (420)	40.32 (3822)

^¥^Among past-year smokers; according to chi-squared analyses, superscript a and b differ *p* < 0.001

**Table 2:** Prevalence of current smoking, ever smoking, and quit attempts among past-year smokers before and after the increase in age-of-sale

	16–17 year olds (*n* = 455 before, *n* = 4735 after), % (*n*)	18–24 year olds (*n* = 2186 before, *n* = 28,495 after), % (*n*)
Current smoker		
Before	23.74 (108)	37.42 (818)
After	14.80 (701)	28.07 (7990)
Ever smoker		
Before	26.59 (334)	43.37 (1238)
After	16.96 (803)	33.35 (9495)
Quit attempt^¥^		
Before	63.25 (74)	50.28 (443)
After	41.38 (305)	38.82 (3251)

^¥^Among past-year smokers

**Table 3:** Primary analysis—results of the GAM analyses fitting linear trends to current smoking status, ever smoking status, and quit attempts among past-year smokers (16–17 versus 18–24 year olds)

	Unadjusted					Adjusted for sex and social grade and population-level policies				
	OR	95%CI		*p*	BFs	OR	95%CI		*p*	BFs
		Lower	Upper				Lower	Upper		
Current smoking										
Trend	0.982	0.956	1.008	0.179		0.979	0.954	1.006	0.127	
Level	0.903	0.670	1.218	0.504		0.807	0.456	1.426	0.460	
Change in slope	1.012	0.985	1.039	0.389		1.012	0.986	1.041	0.364	
Age *(16–17 ref)*	1.922	1.523	2.426	< 0.001		1.953	1.542	2.475	< 0.001	
Level × age	1.113	0.838	1.378	0.458	0.80	1.103	0.827	1.472	0.504	0.75
Slope × age	1.001	0.999	1.004	0.233	1.22	1.002	0.999	1.004	0.141	2.10
Ever smoking										
Trend	0.981	0.955	1.007	0.145		0.978	0.953	1.004	0.103	
Level	0.950	0.713	1.267	0.727		0.899	0.520	1.552	0.702	
Change in slope	1.012	0.986	1.039	0.358		1.012	0.985	1.040	0.374	
Age *(16–17 ref)*	2.116	1.690	2.649	< 0.001		2.139	1.703	2.686	< 0.001	
Level × age	1.044	0.785	1.371	0.755	0.52	1.036	0.786	1.366	0.803	0.50
Slope × age	1.002	1.000	1.005	0.040	2.10	1.003	1.000	1.005	0.019	4.20
*Trend after 16–17*	0.993	0.942	1.046			0.990	0.939	1.044		
*Trend after 18–24*	0.995	0.942	1.052			0.993	0.939	1.049		
Quit attempts										
Trend	0.985	0.945	1.027	0.485		0.984	0.944	1.027	0.464	
Level	0.529	0.322	0.902	0.019		0.311	0.117	0.825	0.019	
Change in slope	1.011	0.970	1.055	0.598		1.014	0.971	1.058	0.526	
Age (*16–17 ref)*	0.587	0.394	0.874	0.009		0.585	0.393	0.872	0.009	
Level × age	1.770	1.081	2.899	0.023	5.68	1.760	1.075	2.884	0.025	5.49
Slope × age	0.998	0.994	1.003	0.440	0.71	0.998	0.994	1.003	0.460	0.71
*Level 16–17*	0.529	0.322	0.902			0.311	0.117	0.825		
*Level 18–24*	0.936	0.348	2.615			0.547	0.126	2.379		

*BFs* Bayes factors; the ORs representing the main effects for level and slope (i.e. *β*_3_level and *β*_4_slope) can be interpreted as the effect among 16–17 year olds, while the effect among the comparison groups is calculated as the main effects multiplied by the ORs for the interactions (i.e. *β*_3_level × (*β*_5_level_*t*_ × age) and *β*_4_slope × (*β*_6_slope × age))

**Table 4:** AIC values for the post-age-of-sale implantation trend analysis (16–17 versus and 18–24 year olds)

	Smoking		Ever smoking		Quit attempts among past-year smokers	
	16–17	18–24	16–17	18–24	16–17	18–24
Linear trend	**25,407**	136,031	**24,867**	133,131	**3653**	**39,621**
Quadratic trend	25,426	**136,025**	24,879	**133,127**	3653	39,621
Cubic trend	25,428	136,026	24,880	133,127	3653	39,621
Linear best fitting segmented	25,408	136,028	24,868	133,129	3655	39,623
Quadratic best fitting segmented	25,413	136,027	24,876	133,129	3655	39,623
Cubic best fitting segmented	25,413	136,028	24,875	133,129	3655	39,634

**Table 5:** Secondary analysis—results of the best fitting GAMM post-age-of-sale trend analysis

	Unadjusted				Adjusted for sex and social grade and population-level policies			
	OR	95%CI		*p*	OR	95%CI		*p*
		Lower	Upper			Lower	Upper	
Current smoking 16–17								
Trend	0.996	0.928	1.070	0.919	1.005	0.935	1.081	0.883
Level	0.841	0.537	1.319	0.452	0.739	0.456	1.199	0.221
Change in slope	0.997	0.929	1.071	0.936	0.988	0.919	1.062	0.743
Current smoking 18–24								
Trend	0.979	0.951	1.007	0.144	0.975	0.947	1.003	0.082
Level	1.073	0.887	1.297	0.468	1.119	0.919	1.362	0.264
Change in slope	1.014	0.985	1.004	0.336	1.017	0.988	1.047	0.253
Change in slope ^2	1.000	1.000	1.000	0.114	**1.000**	**1.000**	**1.000**	**0.003**
Ever smoking 16–17								
Trend	0.989	0.922	1.061	0.756	0.996	0.928	1.070	0.914
Level	0.915	0.588	1.423	0.694	0.844	0.526	1.356	0.484
Change in slope	1.004	0.936	1.077	0.912	0.997	0.928	1.070	0.928
Ever smoking 18–24								
Trend	0.979	0.952	1.007	0.133	0.974	0.947	1.002	0.073
Level	1.030	0.857	1.240	0.751	1.059	0.874	1.284	0.556
Change in slope	1.016	0.988	1.045	0.278	1.019	0.990	1.048	0.196
Change in slope ^2	1.000	1.000	1.000	0.377	**1.000**	**1.000**	**1.000**	**0.029**
Quit attempts 16–17								
Trend	1.019	0.902	1.151	0.766	1.028	0.907	1.164	0.666
Level	**0.451**	**0.204**	**1.000**	**0.050**	0.448	0.193	1.036	0.061
Change in slope	0.978	0.867	1.105	0.723	0.969	0.855	1.098	0.623
Change in slope 18–24								
Trend	0.982	0.939	1.026	0.412	0.980	0.937	1.024	0.364
Level	0.975	0.737	1.290	0.859	0.982	0.735	1.311	0.901
Change in slope	1.013	0.969	1.059	0.561	1.016	0.971	1.062	0.497

Finally, additional sensitivity analyses were run following reviewer comments which (1) adjusted for longer-term step-level effects for the two policies occurring closest in time to the increase in age-of-sale: the introduction of a ban in enclosed public spaces and the pictorial health warnings on product packaging (long-term step-level effects were not assessed for the other policies due to model overparameterisation and lack of power) and (2) adjusted for self-reported weekly spend on tobacco. See [38, 39] for a detailed introduction to Kalman filtering. Additional file 1: Table S8 gives the results from these analyses. Findings were similar as those for the pre-planned primary analysis.

## Results

**Fig. 1:**
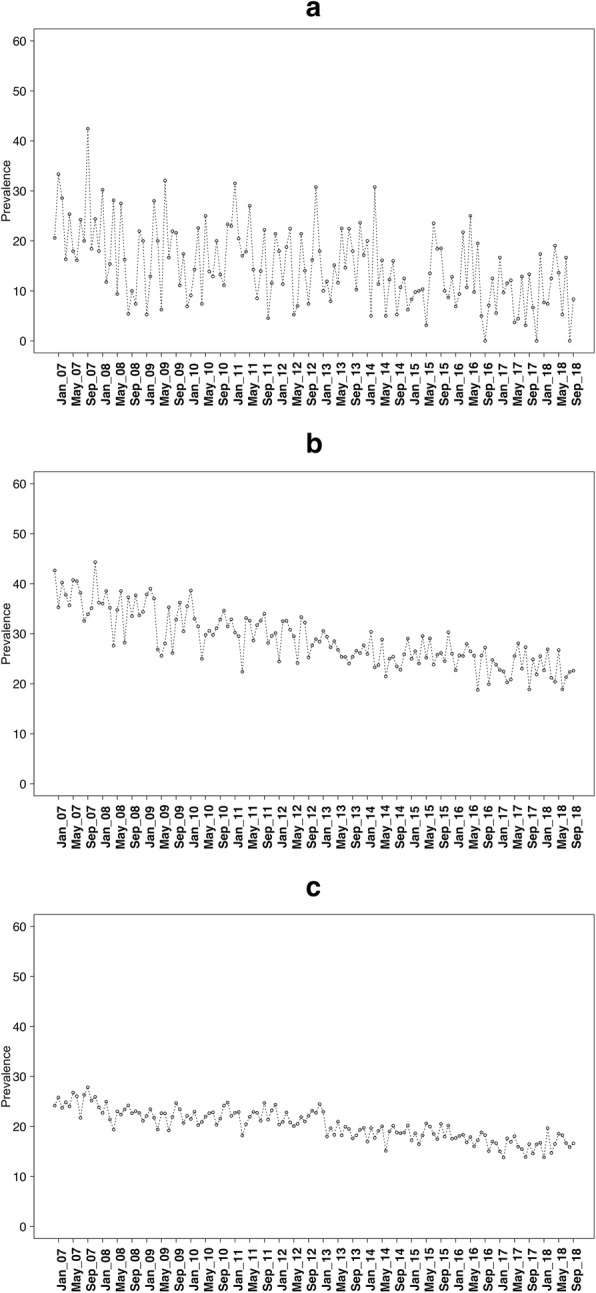
Smoking prevalence for **a** 16–17 year olds, **b** 18–24 year olds, and **c** 25+ year olds

**Fig. 2:**
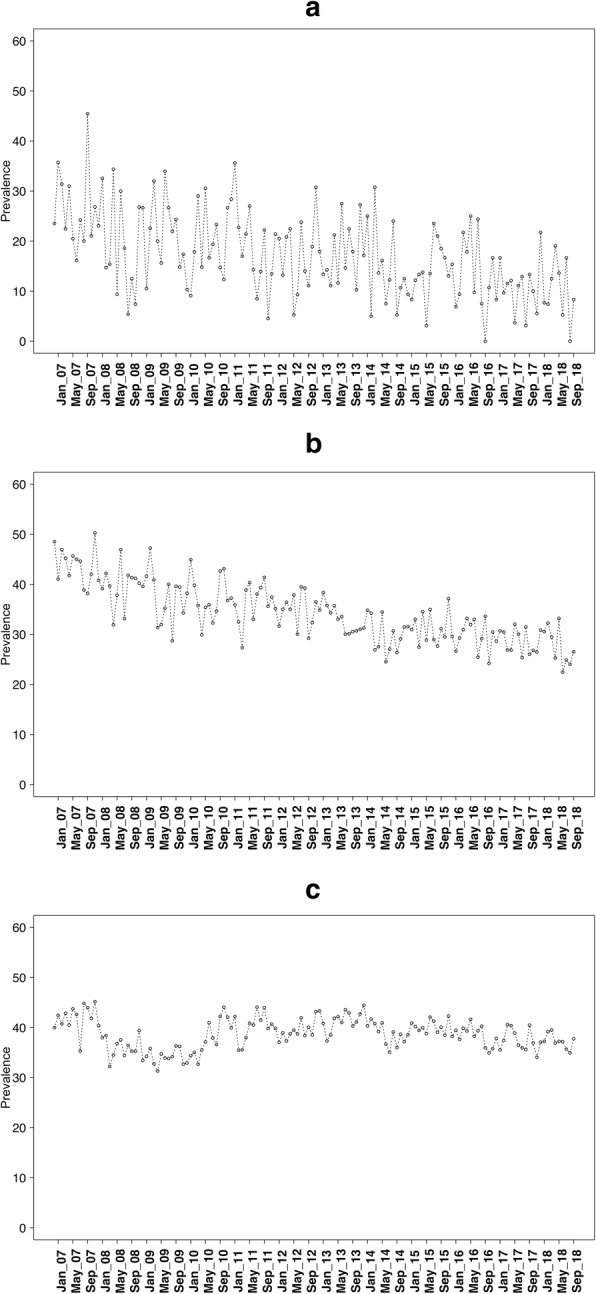
Ever smoking prevalence for **a** 16–17 year olds, **b** 18–24 year olds, and **c** 25+ year olds

**Fig. 3:**
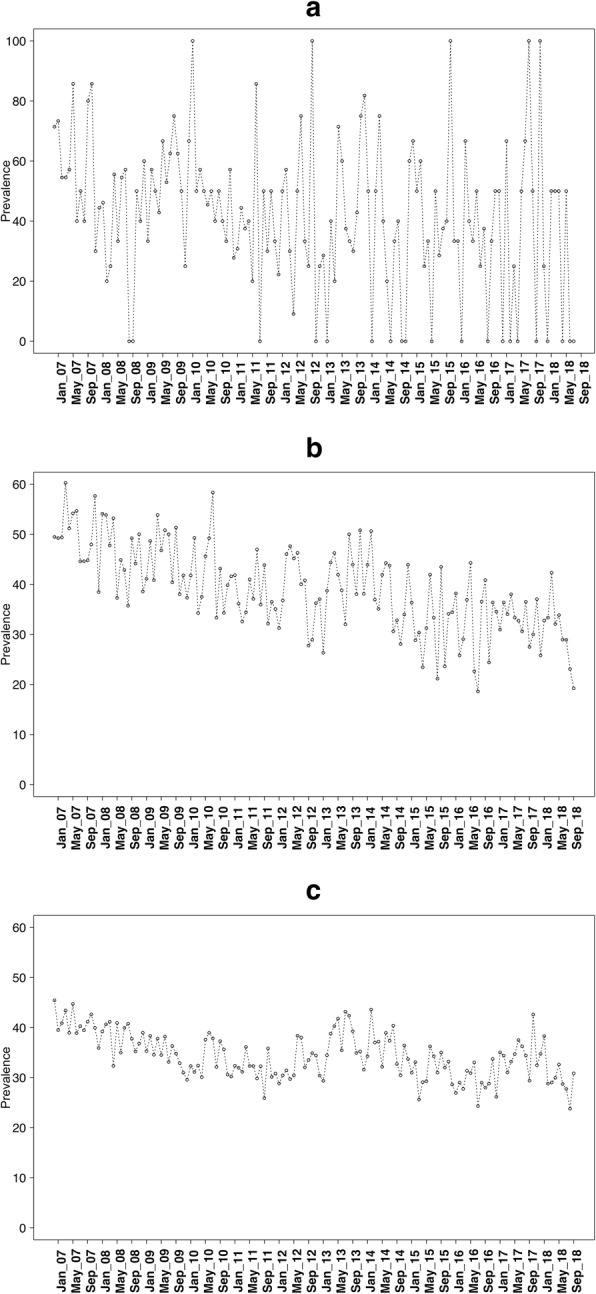
Prevalence of quit attempts for **a** 16–17 year olds, **b** 18–24 year olds, and **c** 25+ year olds

Data were collected on 252,601 adults aged 16+ between November 2006 and September 2018. Of these, 2.06% (95% CI 2.01 to 2.12) were aged 16–17, 12.19% (95% CI 12.06 to 12.31) were aged 18–24, and 85.75% (95% CI 85.62 to 85.90) were aged 25+ years. Figures 1, 2, and 3 show the prevalence of current smoking, ever smoking, and quit attempts over time in these three age groups.

Table 1 gives descriptive statistics for the sample as a whole and as function of the two age groups of interest from the pre-registered analysis (results for those aged 25+ can be found in Additional file 1: Table S1–S5 and summarised under unplanned analyses). Compared with the group aged 18–24 years, 16–17 year olds reported lower prevalence of current smoking and ever smoking and (among past-year smokers) higher prevalence of quit attempts.

### Primary analysis—difference-in-differences

Table 2 shows the prevalence of current smoking, ever smoking, and quit attempts among past-year smokers before and after the increase in age-of-sale as a function of age. Table 3 shows the results of the primary analysis.

Following the rise in age-of-sale, there were no significant step-level changes in either current smoking or ever smoking. However, for ever smoking, a greater declining trend was found among 16–17 year olds relative to 18–24 year olds following the increase in age-of-sale. For every post-implementation month, the odds of ever smoking decreased by 1% for those aged 16–17 and 0.7% for those aged 18–24 (OR 0.990 versus 0.993, *p* = 0.019). For quit attempts only, there was also a significantly greater step-level decline among 16–17 year olds relative to 18–24 year olds after the increase in age-of-sale. The overall odds of a quit attempt decreased by 69% among those aged 16–17 and 45% among those aged 18–24 (OR 0.311 versus 0.547, *p* = 0.025) following the increase in age-of-sale implementation. Bayes factors indicated that the data were largely insensitive to detect other associations. Additional file 1: Fig. S1 shows the results of the fitted GAM models.

### Secondary analysis—trend analysis

Whereas the trends for current smoking and ever smoking post-increase in age-of-sale were linear for 16–17 year olds, they were better explained by a quadratic function (slowing declines) for 18–24 year olds. Trends in quit attempts post-increase in age-of-sale followed a linear decline across both age groups (see Tables 4 and 5).

### Sensitivity analyses—comparison with 18–21 and 22–24 year olds

As for the comparison with 18–24 year olds, there was a significantly greater declining trend in ever smoking among 16–17 year olds following the increase in age-of-sale compared with 22–24 year olds but not relative to 18–21 year olds (Additional file 1: Table S6–S7). As before, a greater step-level decline in quit attempts was found for 16–17 year olds versus 18–21 year olds (Additional file 1: Table S6) and 22–24 year olds (Additional file 1: Table S7). All other associations were non-significant.

### Unplanned analyses—comparison with 25+ year olds

Additional file 1: Table S1 gives descriptive statistics for the sample as a whole and as function of the two age groups of interest (16–17 versus 25+ year olds). Additional file 1: Table S2 shows the prevalence of smoking, ever smoking, and quit attempts among past-year smokers before and after the increase in age-of-sale as a function of age, while Additional file 1: Table S3 shows the results of the primary analysis.

Following the increase in age-of-sale, there was a step-level decline in current smoking across both age groups (OR 0.756, *p* = 0.047) but no significant step-level change in ever smoking (OR 0.847, *p* = 0.216). This was then followed by a larger declining trend in current smoking among 16–17 year olds relative to 25+ year olds (OR 0.994 versus 0.998, *p* = 0.002). There was also a decline in ever smoking among 16–17 year olds over time but an increase among 25+ year olds (OR 0.993 versus 1.001, *p* < 0.001). In contrast, although the probability of making a quit attempt remained higher among 16–17 year olds across the study period, there was a significantly greater step-level decline relative to 25+ year olds after the increase in age-of-sale. Bayes factors indicated that the data were largely insensitive to detect other associations. There was evidence for the null hypothesis of no difference between age groups in a step-level change for ever smoking. Additional file 1: Fig. 1 shows the results of the fitted GAM models.

The post-increase in age-of-sale trends for smoking, ever smoking, and quit attempts was linear for 16–17 year olds and 25+ year olds Additional file 1: Table S4-S5.

## Discussion

### Statement of principal findings

To our knowledge, this is the first attempt to examine the long-term impact of the increase in age-of-sale of cigarettes from 16 to 18 years in England on smoking behaviour. The results indicate that although ever smoking declined across age groups in the decade following the increase in age-of-sale, the decline was greater among 16–17 year olds compared with 18–24 year olds. In contrast, a greater step-level decline in quit attempts was also found among 16–17 year olds. These findings were largely corroborated in the comparison with those aged 25+, where a greater post-implementation decline was also found in current smoking among 16–17 year olds compared with 25+ year olds.

### Comparison with previous studies

These findings are consistent with previous studies in England showing a short-term positive impact of increasing the legal age-of-sale [2, 6]. In the study by Fidler and West [2], the odds of smoking were 36% higher for those aged 18+ compared to 16 to 17 year olds over the 18-month period following the increase in age-of-sale. In this study, for every post-implementation month, the odds of ever smoking were around 0.3% lower for those aged 16–17 compared to 18–24 year olds and 0.8% lower compared to those aged 25+, thus a difference in odds of about 5.4% and 14.4% over the same time period as Fidler and West. This would suggest a significant but smaller impact long term. The larger decline in quit attempts among 16–17 year olds than 18–24 year olds may reflect a temporary increase in quitting activity occurring just before the increase in age-of-sale as a consequence of ‘smokefree’ legislation which was implemented in July 2007 [18]. Alternatively, it could reflect a change in the characteristics of smokers over time (e.g. they become more dependent and less motivated to quit). Previous studies have found that motivation to quit is a major predictor of quit attempts at the individual and population level [40, 41]. However, there is little support for the ‘hardening hypothesis’ [42]. Although motivation to quit appears to have declined among smokers, nicotine dependence is decreasing [43].

### Strengths and weaknesses of the study

There are several limitations of the current study which need to be considered. First, it was not possible to obtain saliva samples to confirm smoking status, and therefore, misreporting may bias results. However, there was no reason to believe that misreporting would vary over time or between age groups. Secondly, due to low power, owing to the small number of waves of data collection before the age-of-sale was implemented, it was not possible to model the pre-implementation trend separately among the age groups or to stratify findings according to socio-demographic characteristics. Thus, we cannot address questions such as whether the smoking behaviour of those in routine and manual social grades was differentially affected relative to those in higher social grades. It is conceivable that those in more disadvantaged groups were less affected by the increase in the age-of-sale because they have greater access to cigarettes from social and family networks and through illicit sources [44]. Thirdly, Bayes factors suggested that for the primary comparison, our study was insensitive to detect a difference in smoking prevalence following the increase in age-of-sale between those aged 16–17 and those aged 18–24. This is likely also due to short pre-implementation trend, which meant that the study was not powered to detect the hypothesised effects. Fourthly, although we adjusted for other population-level policies and tobacco control mass media spend, we only considered temporary 1-month pulse effects and step level changes for the two policies occuring closest in time. It is possible that any impact may have been in a different form, e.g. a change in trend. This warrants further investigation but was beyond the scope of the current study and would have required substantially more power. Fifthly, although we a priori decided on the covariates to adjust for in the current study in order to prevent overparameterisation, there are several unadjusted confounders that should be considered. For example, although cost of cigarettes increases linearity over time and is therefore accounted for by the underlying linear trend, and there does not appear to be an association between the affordability of cigarettes and quitting activity [14], tax increases have been shown to impact on youth smoking rates [33]. However, the effectiveness of tax increases is often undermined by the use of ‘undershifting’ (absorbing the tax increases) by tobacco companies [45]. Finally, this study’s measure of socio-economic status, social grade, has limitations for young adults as it is based on the occupation of the chief income earner [15, 46]. However, to prevent overparameterisation and multicollinearity, it was agreed a priori that we should only adjust for one socio-economic measure. This measure in the STS has been found to have the strongest independent association with smoking status among people in England while adjusting for age [47]. The important feature of covariates for trend analyses is that they pick up any systematic changes in a population over time, rather than their categorisation accuracy.

### Implications

The current study adds to previous research that has documented an association between the change in age-of-sale and a drop in youth smoking [2, 6], and supports the view that such legislation may be an effective policy for tobacco control. Given the potential impact on the future health of a generation of young adults, it raises the question as to whether we should increase the legal age-of-sale even further to 21 years. Central to the original argument for increasing the age-of-sale from 16 to 18 was that the majority of smokers had tried their first cigarette by this age [48]. However, smokers’ transition to regular daily use generally occurs between 18 and 21 years of age [49]. Tobacco companies have also long viewed young adults aged 18 to 21 as a key target market group [50]. A hypothetical health policy model in which the tobacco age-of-sale was increased to 21 years has projected that youth smoking prevalence could be expected to drop by over 10% within 7 years [51]. Currently, the WHO Framework Convention on Tobacco Control (FCTC) specifically supports measures to prohibit the sales of tobacco products to those classed as ‘minors’ as set by national law or the age of 18, but also encourages parties to the FCTC to implement measures beyond those required by the convention [52].

## Conclusion

In conclusion, the increase in legal age-of-sale of cigarettes from 16 to 18 years in England was associated with a greater long-term (≥ 10 year) decline in ever smoking among those aged 16–17 compared with those aged 18–24 years. Data on current smoking and quit attempts were inconclusive for this comparison.

## Supplementary information

**Additional file 1: Figure S1.** Fitted model for (a) smoking status (b) ever smoking status and (c) attempts to quit smoking for 16-17 year olds versus 18-24 year olds and (d) smoking status (e) ever smoking status and (f) attempts to quit smoking for 16-17 year olds versus 25+ year olds. **Supplementary Table 1**. Descriptive statistics of the sample overall as a function of the two age groups of interest (16-17 year olds and 25+ year olds). **Supplementary Table 2**. Prevalence of current smoking, ever smoking, and quit attempts among past year smokers before and after the increase in age-of-sale. **Supplementary Table 3**. Primary analysis - results of the GAM analyses fitting linear trends to current smoking status, ever smoking status and quit attempts among past year smokers (16-17 versus 25+ year olds). **Supplementary Table 4**: AIC values for the post age-of-sale implantation trend analysis (16-17 versus and 25+ year olds). **Supplementary Table 5**: Secondary analysis - results of the best fitting GAMM post-age-of-sale trend analysis. **Supplementary Table 6**: Sensitivity analysis results of the GAM analyses fitting linear trends to current smoking status, ever smoking status and quit attempts among past year smokers among 16-17 year olds and 18-21 year olds. **Supplementary Table 7**: Sensitivity analysis results of the GAM analyses fitting linear trends to current smoking status, ever smoking status and quit attempts among past year smokers among 16-17 year olds and 22-24 year olds. **Supplementary Table 8**: Sensitivity analysis - results of the GAM analyses fitting linear trends to current smoking status, ever smoking status and quit attempts among past year smokers (16-17 versus 18-24 year olds) with additional adjustments.

## Data Availability

For access to the Smoking Toolkit Study, please contact JB or EB (jamie.brown@ucl.ac.uk and e.beard@ucl.ac.uk). The R code for this paper is available on request from EB.

## Abbreviations

GAMs: Generalised Additive Models
STS: Smoking Toolkit Study
