# Supplementary material for: Long-term evaluation of the rise in legal age-of-sale of cigarettes from 16 to 18 in England: a trend analysis
Source: BMC Med. 2020 Apr 8;18:85. doi: 10.1186/s12916-020-01541-w (PMC7140583; doi:10.1186/s12916-020-01541-w)
Supplement: Supplementary file 1 — Additional file 1: Figure S1. Fitted model for (a) smoking status (b) ever smoking status and (c) attempts to quit smoking for 16-17 year olds versus 18-24 year olds and (d) smoking status (e) ever smoking status and (f) attempts to quit smoking for 16-17 year olds versus 25+ year olds. Supplementary Table 1. Descriptive statistics of the sample overall as a function of the two age groups of interest (16-17 year olds and 25+ year olds). Supplementary Table 2. Prevalence of current smoking, ever smoking, and quit attempts among past year smokers before and after the increase in age-of-sale. Supplementary Table 3. Primary analysis - results of the GAM analyses fitting linear trends to current smoking status, ever smoking status and quit attempts among past year smokers (16-17 versus 25+ year olds). Supplementary Table 4: AIC values for the post age-of-sale implantation trend analysis (16-17 versus and 25+ year olds). Supplementary Table 5: Secondary analysis - results of the best fitting GAMM post-age-of-sale trend analysis. Supplementary Table 6: Sensitivity analysis results of the GAM analyses fitting linear trends to current smoking status, ever smoking status and quit attempts among past year smokers among 16-17 year olds and 18-21 year olds. Supplementary Table 7: Sensitivity analysis results of the GAM analyses fitting linear trends to current smoking status, ever smoking status and quit attempts among past year smokers among 16-17 year olds and 22-24 year olds. Supplementary Table 8: Sensitivity analysis - results of the GAM analyses fitting linear trends to current smoking status, ever smoking status and quit attempts among past year smokers (16-17 versus 18-24 year olds) with additional adjustments. [file 12916_2020_1541_MOESM1_ESM.docx]

**Supplementary Table 1: Descriptive statistics of the sample overall as a function of the two age groups of interest (16-17 year olds and 25+ year olds)**

|  | **Overall**  **(n=252,601)**  **%(n)** | **16-17 year olds (n=5,190)**  **%(n)** | **25+ year olds (n=215,911)**  **%(n)** |
| --- | --- | --- | --- |
| **Gender**  *Female*  *Other* | 51.64 (130,434)  48.36 (122,167) | 42.91(2,227)^a^  57.09(2,962) | 49.08 (23,178)^ç^  50.82 (24,051) |
| **Social-grade**  *ABC1*  *C2DE* | 60.72 (178,191)  29.28 (74,410) | 64.89(3,369)^a^  35.09(1,821) | 58.49 (27,621)^ç^  41.52 (19,608) |
| **Current smoker** | 21.33 (53,831) | 15.59(809)^a^ | 20.45 (44,120)^ç^ |
| **Ever smoker** | 37.66 (95,060) | 17.81(924)^a^ | 38.70 (83,492) ^ç^ |
| **Quit attempt^¥^** | 35.87 (20,238) | 44.38(379)^a^ | 34.70 (16,141) ^ç^ |

Note: **^¥^**Among past year smokers; according to chi-squared analyses a and b differ p<0.001.

**Supplementary Table 2: Prevalence of current smoking, ever smoking, and quit attempts among past year smokers before and after the increase in age-of-sale**

|  | **16-17 year olds (n=455 before n=4,735 after)**  **%(n)** | **25+ year olds (n=17,885 before n=198,026 after)**  **%(n)** |
| --- | --- | --- |
| **Current smoker** |  |  |
| Before  After | 23.74 (108)  14.80 (701) | 25.05 (4479)  20.03 (39641) |
| **Ever smoker** |  |  |
| Before  After | 26.59 (334)  16.96 (803) | 41.61 (10,440)  38.43 (76,051) |
| **Quit attempt^¥^** |  |  |
| Before  After | 63.25 (74)  41.38 (305) | 41.25 (1,988)  33.94 (14,153) |

Note: **^¥^**Among past year smokers

**Supplementary Table 3: Primary analysis - results of the GAM analyses fitting linear trends to current smoking status, ever smoking status and quit attempts among past year smokers (16-17 versus 25+ year olds)**

|  |  | **Unadjusted** | |  |  |  | **Adjusted for sex and social-grade and population level policies** | |  |  |
| --- | --- | --- | --- | --- | --- | --- | --- | --- | --- | --- |
|  |  | **95%CI** | |  |  |  | **95%CI** | |  |  |
|  | **OR** | **Lower** | **Upper** | **P** | **BFs** | **OR** | **Lower** | **Upper** | **P** | **BFs** |
| Current smoking |  |  |  |  |  |  |  |  |  |  |
| Trend | 1.005 | 0.994 | 1.017 | 0.349 |  | 1.010 | 0.998 | 1.022 | 0.109 |  |
| Level | 0.794 | 0.605 | 1.041 | 0.095 |  | 0.756 | 0.573 | 0.997 | 0.047 |  |
| Change in slope | 0.988 | 0.977 | 1.000 | 0.044 |  | 0.984 | 0.972 | 0.996 | 0.009 |  |
| Age *(16-17 ref)* | 1.073 | 0.862 | 1.335 | 0.527 |  | 1.131 | 0.906 | 1.412 | 0.275 |  |
| Level*Age | 1.157 | 0.886 | 1.513 | 0.284 | 0.84 | 1.144 | 0.872 | 1.502 | 0.332 | 0.75 |
| Slope*Age | 1.003 | 1.000 | 1.005 | 0.018 | 4.20 | 1.004 | 1.001 | 1.006 | 0.002 | 10.08 |
| *Trend after 16-17* | 0.993 | 0.971 | 1.017 |  |  | 0.994 | 0.970 | 1.018 |  |  |
| *Trend after 25+* | 0.996 | 0.971 | 1.022 |  |  | 0.998 | 0.971 | 1.024 |  |  |
| Ever smoking |  |  |  |  |  |  |  |  |  |  |
| Trend | 1.003 | 0.992 | 1.015 | 0.522 |  | 1.004 | 0.994 | 1.015 | 0.400 |  |
| Level | 0.844 | 0.640 | 1.113 | 0.198 |  | 0.847 | 0.652 | 1.101 | 0.216 |  |
| Change in slope | 0.990 | 0.978 | 1.002 | 0.047 |  | 0.989 | 0.978 | 0.999 | 0.033 |  |
| Age *(16-17 ref)* | 1.967 | 1.576 | 2.454 | <0.001 |  | 2.127 | 1.721 | 2.629 | <0.001 |  |
| Level*Age | 0.980 | 0.643 | 1.008 | 0.979 | 0.22 | 0.964 | 0.745 | 1.248 | 0.783 | 0.23 |
| Slope*Age | 1.008 | 1.006 | 1.010 | <0.001 | >100 | 1.008 | 1.006 | 1.011 | <0.001 | >100 |
| *Trend after 16-17* | 0.993 | 0.970 | 1.017 |  |  | 0.993 | 0.972 | 1.014 |  |  |
| *Trend after 25+* | 1.001 | 0.976 | 1.027 |  |  | 1.001 | 0.978 | 1.025 |  |  |
| Quit attempts |  |  |  |  |  |  |  |  |  |  |
| Trend | 0.989 | 0.970 | 1.008 | 0.254 |  | 0.991 | 0.972 | 1.011 | 0.377 |  |
| Level | 0.529 | 0.328 | 0.853 | 0.009 |  | 0.565 | 0.349 | 0.912 | 0.020 |  |
| Change in slope | 1.008 | 0.988 | 1.028 | 0.450 |  | 1.005 | 0.985 | 1.026 | 0.603 |  |
| Age (*16-17 ref)* | 0.407 | 0.279 | 0.596 | <0.001 |  | 0l.405 | 0.277 | 0.593 | <0.001 |  |
| Level*Age | 1.645 | 1.026 | 2.638 | 0.039 | 4.79 | 1.632 | 1.017 | 2.618 | 0.042 | 4.51 |
| Slope*Age | 1.002 | 0.997 | 1.006 | 0.443 | 1.24 | 1.002 | 0.997 | 1.006 | 0.447 | 1.24 |
| *Level 16-17* | 0.529 | 0.328 | 0.853 |  |  | 0.565 | 0.349 | 0.912 |  |  |
| *Level 25+* | 0.870 | 0.337 | 2.250 |  |  | 0.922 | 0.355 | 2.388 |  |  |

Note: BFs = Bayes Factors; The ORs representing the main effects for level and slope (i.e. $\beta_{3}level and \beta_{4}slope)$ can be interpreted as the effect among 16-17 year olds; while the effect among the comparison groups is calculated as the main effects multiplied by the ORs for the interactions (i.e. $\beta_{3}level*$ ${(\beta}_{5}{level}_{t}*age) and {\beta_{4}slope*(\beta}_{6}slope *age))$

**Supplementary Table 4: AIC values for the post age-of-sale implantation trend analysis (16-17 versus and 25+ year olds)**

|  |  |  |  |  |  |  |
| --- | --- | --- | --- | --- | --- | --- |
|  | **Smoking** | | **Ever smoking** | | **Quit attempts among past year smokers** | |
|  | 16-17 | 25+ | 16-17 | 25+ | 16-17 | 25+ |
| Linear trend | 25407 | 1005926 | 24867 | 922838 | 3653 | 43869 |
| Quadratic trend | 25426 | 1006025 | 24879 | 923019 | 3653 | 43869 |
| Cubic Trend | 25428 | 1006008 | 24880 | 923027 | 3653 | 43868 |
| Linear Best fitting segmented | 25408 | 1005964 | 24868 | 923216 | 3655 | 43882 |
| Quadratic Best fitting segmented | 25413 | 1005962 | 24876 | 923157 | 3655 | 43883 |
| Cubic Best fitting segmented | 25413 | 1005958 | 24875 | 923213 | 3655 | 43886 |

**Supplementary Table 5: Secondary analysis - results of the best fitting GAMM post-age-of-sale trend analysis**

|  |  | **Unadjusted** | |  |  | **Adjusted for sex and social-grade and population level policies** | |  |  |
| --- | --- | --- | --- | --- | --- | --- | --- | --- | --- |
|  |  | **95%CI** | |  |  | **95%CI** | |  |  |
|  | OR | Lower | Upper | P | OR | Lower | Upper | P |  |
| **Current smoking 16-17** |  |  |  |  |  |  |  |  |  |
| Trend | 0.996 | 0.928 | 1.070 | 0.919 | 1.005 | 0.935 | 1.081 | 0.883 |  |
| Level | 0.841 | 0.537 | 1.319 | 0.452 | 0.739 | 0.456 | 1.199 | 0.221 |  |
| Change in slope | 0.997 | 0.929 | 1.071 | 0.936 | 0.988 | 0.919 | 1.062 | 0.743 |  |
| **Current smoking** **25+** |  |  |  |  |  |  |  |  |  |
| Trend | 1.005 | 0.994 | 1.017 | 0.365 | 1.009 | 0.997 | 1.021 | 0.129 |  |
| Level | **0.919** | **0.856** | **0.988** | **0.022** | **0.875** | **0.811** | **0.944** | **0.001** |  |
| Change in slope | 0.991 | 0.979 | 1.002 | 0.124 | **0.988** | **0.976** | **1.000** | **0.042** |  |
| **Ever smoking 16-17** |  |  |  |  |  |  |  |  |  |
| Trend | 0.989 | 0.922 | 1.061 | 0.756 | 0.996 | 0.928 | 1.070 | 0.914 |  |
| Level | 0.915 | 0.588 | 1.423 | 0.694 | 0.844 | 0.526 | 1.356 | 0.484 |  |
| Change in slope | 1.004 | 0.936 | 1.077 | 0.912 | 0.997 | 0.928 | 1.070 | 0.928 |  |
| **Ever smoking** **25+** |  |  |  |  |  |  |  |  |  |
| Trend | 1.003 | 0.993 | 1.014 | 0.513 | 1.004 | 0.994 | 1.015 | 0.395 |  |
| Level | **0.827** | **0.775** | **0.881** | **<0.001** | **0.822** | **0.770** | **0.878** | **<0.001** |  |
| Change in slope | 0.997 | 0.987 | 1.007 | 0.582 | 0.996 | 0.986 | 1.007 | 0.502 |  |
| **Quit attempts 16-17** |  |  |  |  |  |  |  |  |  |
| Trend | 1.019 | 0.902 | 1.151 | 0.766 | 1.028 | 0.907 | 1.164 | 0.666 |  |
| Level | **0.451** | **0.204** | **1.000** | **0.050** | 0.448 | 0.193 | 1.036 | 0.061 |  |
| Change in slope | 0.978 | 0.867 | 1.105 | 0.723 | 0.969 | 0.855 | 1.098 | 0.623 |  |
| **Quit attempts 25+** |  |  |  |  |  |  |  |  |  |
| Trend | 0.998 | 0.969 | 1.008 | 0.237 | 0.990 | 0.971 | 1.010 | 0.340 |  |
| Level | **0.873** | **0.773** | **0.986** | **0.029** | 0.924 | 0.813 | 1.049 | 0.221 |  |
| Change in slope | 1.010 | 0.990 | 1.030 | 0.322 | 1.008 | 0.988 | 1.028 | 0.435 |  |

**Supplementary Table 6: Sensitivity analysis results of the GAM analyses fitting linear trends to current smoking status, ever smoking status and quit attempts among past year smokers among 16-17 year olds and 18-21 year olds**

|  |  | **Unadjusted** | |  |  | **Adjusted for sex and social-grade and population level policies** | |  |
| --- | --- | --- | --- | --- | --- | --- | --- | --- |
|  |  | **95%CI** | |  |  | **95%CI** | |  |
|  | **OR** | **Lower** | **Upper** | **P** | **OR** | **Lower** | **Upper** | **P** |
| **Current smoking** |  |  |  |  |  |  |  |  |
| Trend | 0.979 | 0.947 | 1.013 | 0.218 | 0.977 | 0.944 | 1.011 | 0.179 |
| Level | 0.804 | 0.453 | 1.428 | 0.457 | 0.806 | 0.448 | 1.452 | 0.474 |
| Slope | 1.014 | 0.981 | 1.049 | 0.408 | 1.017 | 0.982 | 1.052 | 0.348 |
| Age *(16-17 ref)* | 1.872 | 1.467 | 2.388 | <0.001 | 1.897 | 1.483 | 2.428 | <0.001 |
| Level*Age | 1.134 | 0.843 | 1.526 | 0.404 | 1.128 | 0.835 | 1.523 | 0.434 |
| Slope*Age | 1.000 | 0.998 | 1.003 | 0.828 | 1.001 | 0.998 | 1.003 | 0.666 |
| **Ever smoking** |  |  |  |  |  |  |  |  |
| Trend | 0.975 | 0.944 | 1.008 | 0.133 | 0.974 | 0.942 | 1.007 | 0.115 |
| Level | 0.934 | 0.538 | 1.623 | 0.810 | 0.905 | 0.515 | 1.592 | 0.729 |
| Slope | 1.017 | 0.984 | 1.051 | 0.318 | 1.018 | 0.985 | 1.053 | 0.286 |
| Age *(16-17 ref)* | 2.022 | 1.598 | 2.559 | <0.001 | 2.038 | 1.606 | 2.587 | <0.001 |
| Level*Age | 1.043 | 0.785 | 1.387 | 0.770 | 1.041 | 0.780 | 1.390 | 0.783 |
| Slope*Age | 1.001 | 0.999 | 1.004 | 0.338 | 1.001 | 0.999 | 1.004 | 0.240 |
| **Quit attempts** |  |  |  |  |  |  |  |  |
| Trend | 0.973 | 0.924 | 1.026 | 0.311 | 0.970 | 0.920 | 1.023 | 0.261 |
| Level | **0.351** | **0.130** | **0.949** | **0.039** | **0.356** | **0.131** | **0.969** | **0.043** |
| Slope | 1.024 | 0.972 | 1.080 | 0.372 | 1.028 | 0.974 | 1.084 | 0.317 |
| Age (*16-17 ref)* | 0.620 | 0.410 | 0.937 | 0.023 | 0.618 | 0.408 | 0.936 | 0.023 |
| Level*Age | 1.637 | 0.982 | 2.730 | 0.059 | 1.628 | 0.975 | 2.716 | 0.062 |
| Slope*Age | 0.999 | 0.995 | 1.004 | 0.750 | 0.999 | 0.995 | 1.004 | 0.812 |

**Supplementary Table 7: Sensitivity analysis results of the GAM analyses fitting linear trends to current smoking status, ever smoking status and quit attempts among past year smokers among 16-17 year olds and 22-24 year olds**

|  |  | **Unadjusted** | |  |  | **Adjusted for sex and social-grade and population level policies** | |  |
| --- | --- | --- | --- | --- | --- | --- | --- | --- |
|  |  | **95%CI** | |  |  | **95%CI** | |  |
|  | **OR** | **Lower** | **Upper** | **P** | **OR** | **Lower** | **Upper** | **P** |
| **Current smoking** |  |  |  |  |  |  |  |  |
| Trend | 0.996 | 0.959 | 1.034 | 0.831 | 0.999 | 0.961 | 1.038 | 0.954 |
| Level | 0.779 | 0.434 | 1.399 | 0.404 | 0.711 | 0.388 | 1.305 | 0.271 |
| Slope | 0.995 | 0.958 | 1.033 | 0.776 | 0.991 | 0.953 | 1.030 | 0.653 |
| Age *(16-17 ref)* | 2.002 | 1.551 | 2.584 | <0.001 | 2.020 | 1.557 | 2.622 | <0.001 |
| Level*Age | 2.082 | 0.795 | 1.473 | 0.618 | 1.076 | 0.785 | 1.156 | 0.650 |
| Slope*Age |  |  |  |  |  |  |  |  |
| **Ever smoking** |  |  |  |  |  |  |  |  |
| Trend | 0.994 | 0.958 | 1.031 | 0.740 | 1.038 | 0.959 | 1.034 | 0.831 |
| Level | 0.857 | 0.489 | 1.504 | 0.591 | 1.305 | 0.461 | 1.474 | 0.515 |
| Slope | 0.995 | 0.959 | 1.032 | 0.792 | 1.030 | 0.956 | 1.031 | 0.696 |
| Age *(16-17 ref)* | 2.265 | 1.770 | 2.899 | <0.001 | 2.622 | 1.779 | 2.942 | <0.001 |
| Level*Age | 1.040 | 0.773 | 1.400 | 0.794 | 1.475 | 0.757 | 1.388 | 0.872 |
| Slope*Age | **1.004** | **1.002** | **1.006** | **0.001** | **1.006** | **1.002** | **1.007** | **<0.001** |
| **Quit attempts** |  |  |  |  |  |  |  |  |
| Trend | 1.006 | 0.948 | 1.068 | 0.843 | 1.011 | 0.952 | 1.074 | 0.722 |
| Level | **0.246** | **0.089** | **0.680** | **0.007** | **0.253** | **0.091** | **0.707** | **0.009** |
| Slope | 0.993 | 0.935 | 1.055 | 0.830 | 0.989 | 0.930 | 1.051 | 0.711 |
| Age (*16-17 ref)* | 0.542 | 0.353 | 0.832 | 0.005 | 0.543 | 0.353 | 0.834 | 0.005 |
| Level*Age | **1.963** | **1.158** | **3.326** | **0.012** | **1.953** | **1.152** | **3.313** | **0.013** |
| Slope*Age | 0.997 | 0.992 | 1.002 | 0.225 | 0.997 | 0.992 | 1.002 | 0.220 |

**Supplementary Table 8: Sensitivity analysis - results of the GAM analyses fitting linear trends to current smoking status, ever smoking status and quit attempts among past year smokers (16-17 versus 18-24 year olds) with additional adjustments**

|  | Adjusted for sex, social-grade and population level policies (with long-term step level changes for the ban in enclosed public spaces and the pictorial health warnings on product packaging; all other policies modelled as pulse effects) | | | | Adjusted for sex, social-grade, population level policies (with long-term step level changes for the ban in enclosed public spaces and the pictorial health warnings on product packaging; all other policies modelled as pulse effects) and cost of smoking | | | |  |
| --- | --- | --- | --- | --- | --- | --- | --- | --- | --- |
|  |  | 95%CI | | 95%CI |  | 95%CI | |  |  |
|  | OR | Lower | Upper | P | OR | Lower | Upper | P | P |
| Current Smoking |  |  |  |  |  |  |  |  |  |
| Trend | 0.988 | 0.953 | 1.024 | 0.500 | 0.988 | 0.957 | 1.041 | 0.924 |  |
| Level | 0.944 | 0.692 | 1.287 | 0.715 | 0.957 | 0.698 | 1.312 | 0.786 |  |
| Change in Slope | 1.006 | 0.971 | 1.043 | 0.732 | 0.996 | 0.955 | 1.039 | 0.849 |  |
| Age *(16-17 ref)* | 1.952 | 1.541 | 2.473 | <0.001 | 1.949 | 1.538 | 2.468 | <0.001 |  |
| Level*Age | 1.109 | 0.831 | 1.479 | 0.484 | 1.112 | 0.833 | 1.484 | 0.472 |  |
| Slope*Age | 1.002 | 0.999 | 1.004 | 0.159 | 1.002 | 0.999 | 1.004 | 0.168 |  |
| Ever smoking |  |  |  |  |  |  |  |  |  |
| Trend | 0.986 | 0.952 | 1.021 | 0.419 | 0.996 | 0.955 | 1.038 | 0.838 |  |
| Level | 0.967 | 0.718 | 1.302 | 0.823 | 0.983 | 0.726 | 1.331 | 0.909 |  |
| Change in Slope | 1.008 | 0.973 | 1.044 | 0.672 | 0.997 | 0.957 | 1.040 | 0.901 |  |
| Age *(16-17 ref)* | 2.140 | 1.04 | 2.687 | <0.001 | 2.136 | 1.701 | 2.682 | <0.001 |  |
| Level*Age | 1.037 | 0.786 | 1.367 | 0.798 | 1.040 | 0.789 | 1.372 | 0.778 |  |
| Slope*Age | 1.003 | 1.000 | 1.005 | 0.020 | 1.003 | 1.000 | 1.005 | 0.022 |  |
| *Trend after 16-17* | 0.994 | 0.926 | 1.066 |  | 0.993 | 0.914 | 1.069 |  |  |
| *Trend after 18-24* | 0.997 | 0.926 | 1.071 |  | 0.996 | 0.914 | 1.074 |  |  |
| Quit attempts |  |  |  |  |  |  |  |  |  |
| Trend | 0.969 | 0.914 | 1.027 | 0.287 | 0.995 | 0.933 | 1.061 | 0.86 |  |
| Level | 0.522 | 0.309 | 0.884 | 0.016 | 0.551 | 0.324 | 0.937 | 0.028 |  |
| Change in slope | 1.028 | 0.970 | 1.090 | 0.348 | 1.001 | 0.939 | 1.068 | 0.966 |  |
| Age (*16-17 ref)* | 0.589 | 0.395 | 0.878 | 0.009 | 0.583 | 0.391 | 0.869 | 0.008 |  |
| Level*Age | 1.748 | 1.067 | 2.864 | 0.027 | 1.777 | 1.084 | 2.914 | 0.023 |  |
| Slope*Age | 0.998 | 0.994 | 1.003 | 0.470 | 0.998 | 0.994 | 1.003 | 0.422 |  |
| *Level 16-17* | 0.522 | 0.309 | 0.884 |  | 0.551 | 0.324 | 0.937 |  |  |
| *Level 18-24* | 0.912 | 0.330 | 2.532 |  | 0.979 | 0.351 | 2.730 |  |  |

Note: The ORs representing the main effects for level and slope (i.e. $\beta_{3}level and \beta_{4}slope)$ can be interpreted as the effect among 16-17 year olds; while the effect among the comparison groups is calculated as the main effects multiplied by the ORs for the interactions (i.e. $\beta_{3}level*$ ${(\beta}_{5}{level}_{t}*age) and {\beta_{4}slope*(\beta}_{6}slope *age)$)


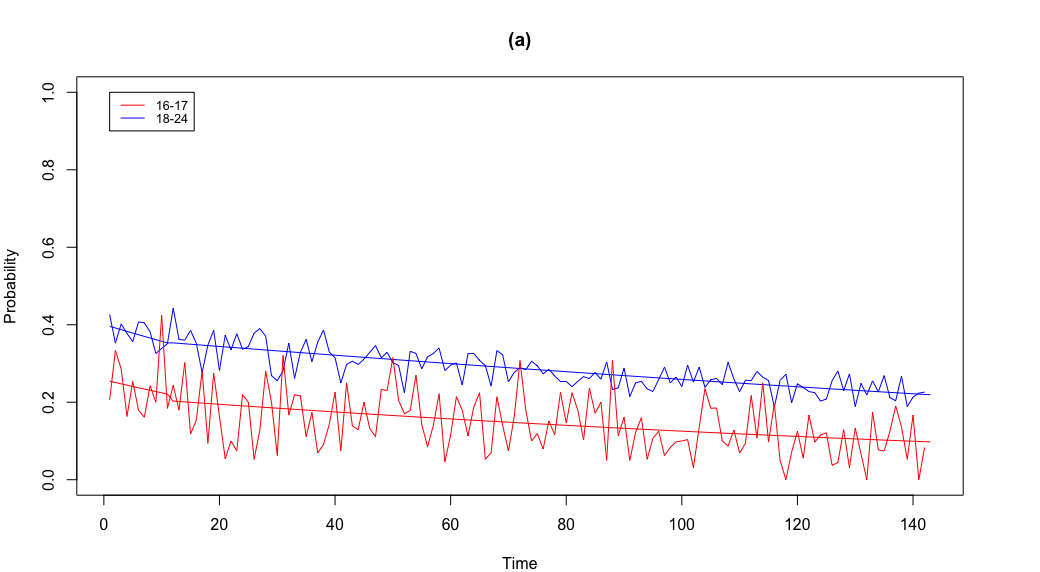


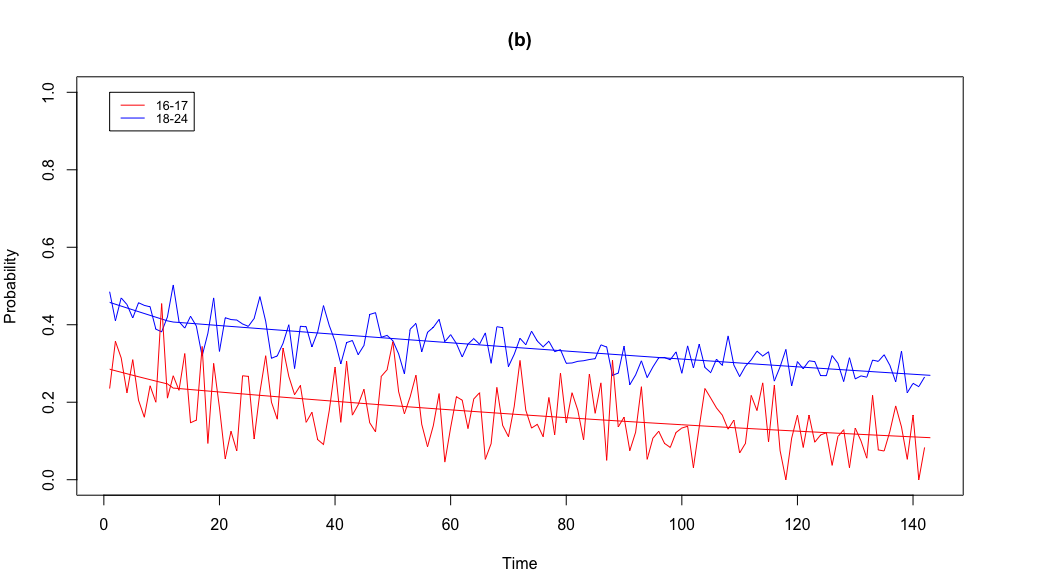


**
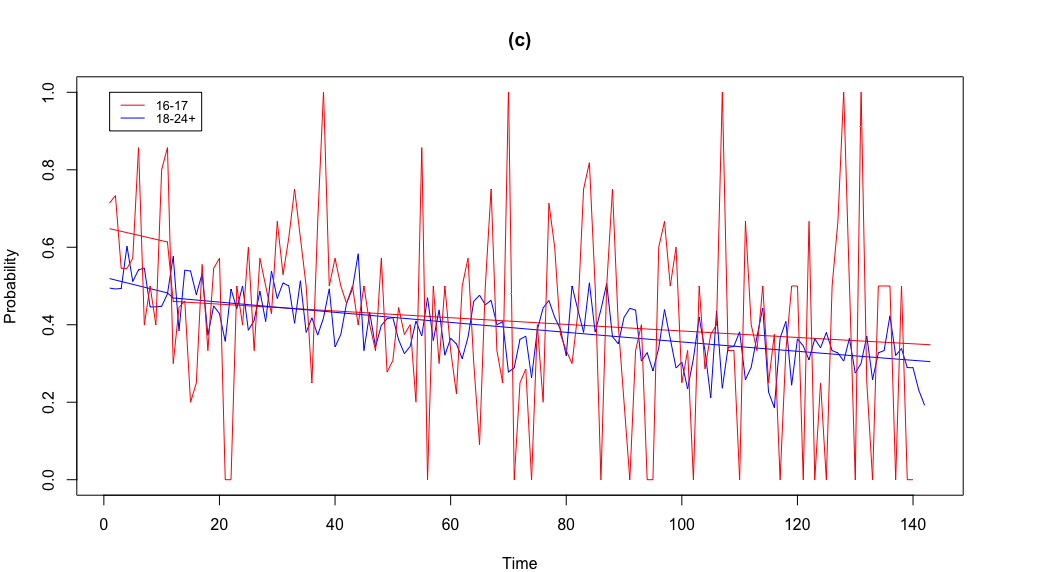
**

**
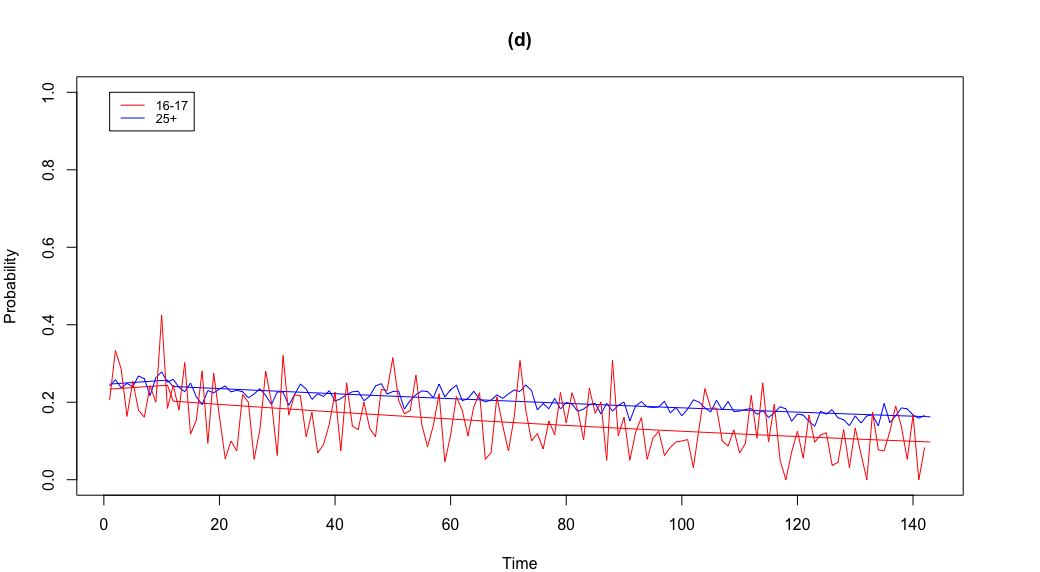
**

**
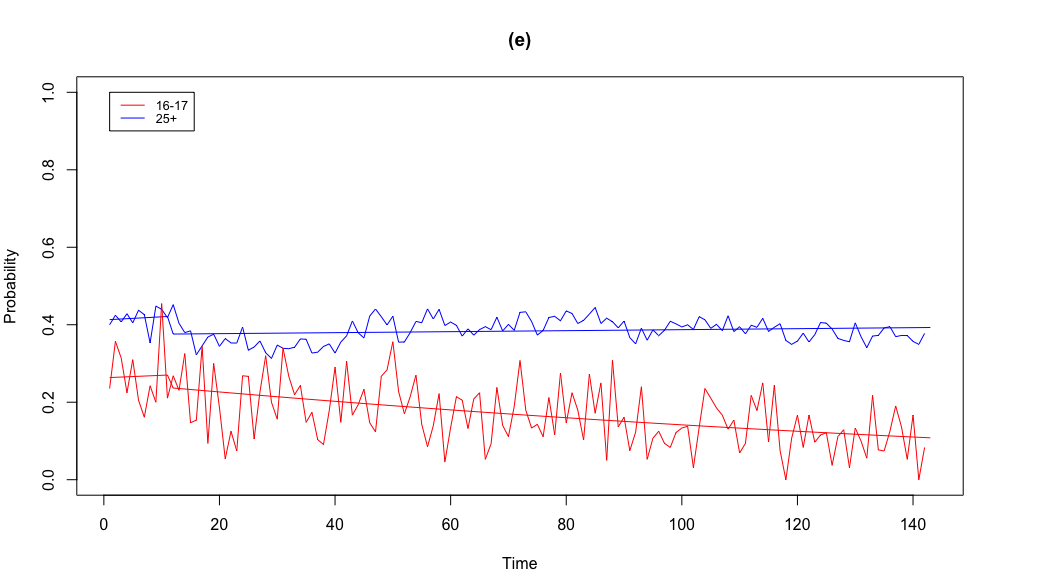
**

**
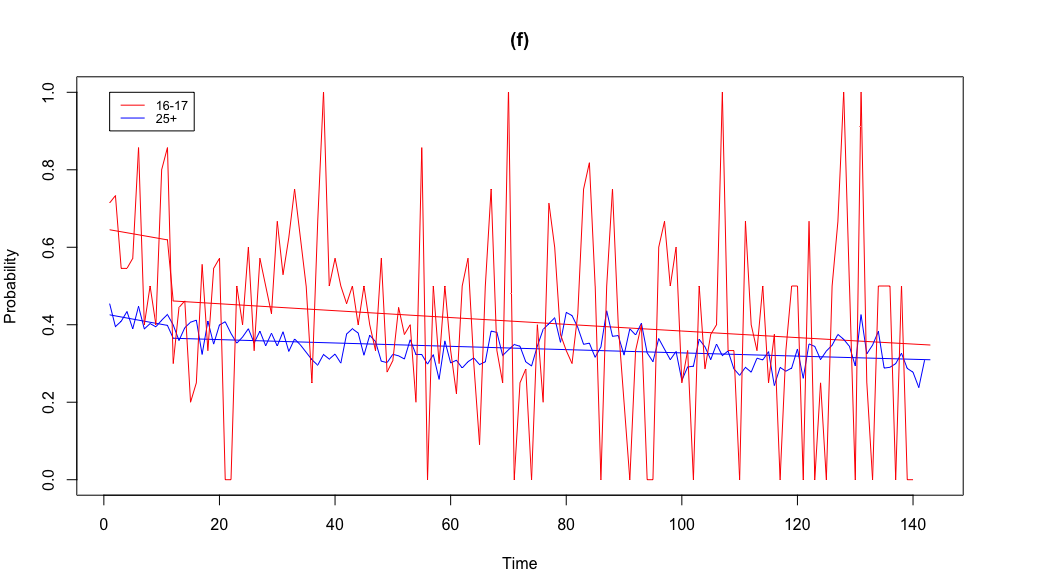
**

**Supplementary Figure 1:** Fitted model for (a) smoking status (b) ever smoking status and (c) attempts to quit smoking for 16-17 year olds versus 18-24 year olds and (d) smoking status (e) ever smoking status and (f) attempts to quit smoking for 16-17 year olds versus 25+ year olds
